# A comparison of ^111^In- or ^64^Cu-DOTA-trastuzumab Fab fragments for imaging subcutaneous HER2-positive tumor xenografts in athymic mice using microSPECT/CT or microPET/CT

**DOI:** 10.1186/2191-219X-1-15

**Published:** 2011-08-17

**Authors:** Conrad Chan, Deborah A Scollard, Kristin McLarty, Serena Smith, Raymond M Reilly

**Affiliations:** 1 Department of Pharmaceutical Sciences, University of Toronto, Toronto, M5S 3M2, ON, Canada; 2 Department of Medical Imaging, University of Toronto, Toronto, M5S 3E2, ON, Canada; 3 Toronto General Research Institute, University Health Network, Toronto, M5G 2M9, ON, Canada

**Keywords:** indium-111, copper-64, HER2, MicroSPECT, MicroPET, DOTA, trastuzumab Fab, breast cancer, ovarian cancer

## Abstract

**Background:**

Our objective was to compare ^111^In- or ^64^Cu-DOTA-trastuzumab Fab fragments for imaging small or large s.c. tumor xenografts in athymic mice that display a wide range of human epidermal growth factor receptor-2 (HER2) expression using microSPECT/CT or microPET/CT.

**Methods:**

Trastuzumab Fab were labeled with ^111^In or ^64^Cu by conjugation to 1,4,7,10-tetraazacyclododecane N, N', N'', N'''-tetraacetic acid (DOTA). The purity of ^111^In- and ^64^Cu-DOTA-trastuzumab Fab was measured by SDS-PAGE and HPLC. HER2 binding affinity was determined in saturation radioligand binding assays using SKBR-3 cells (1.3 × 10^6 ^HER2/cell). MicroSPECT/CT and microPET/CT were performed in athymic mice bearing s.c. BT-20 and MDA-MB-231 xenografts with low (0.5 to 1.6 × 10^5 ^receptors/cell), MDA-MB-361 tumors with intermediate (5.1 × 10^5 ^receptors/cell) or SKOV-3 xenografts with high HER2 expression (1.2 × 10^6 ^receptors/cell) at 24 h p.i. of 70 MBq (10 μg) of ^111^In-DOTA-trastuzumab Fab or 22 MBq (10 μg) of ^64^Cu-DOTA-trastuzumab Fab or irrelevant ^111^In- or ^64^Cu-DOTA-rituximab Fab. Tumor and normal tissue uptake were quantified in biodistribution studies.

**Results:**

^111^In- and ^64^Cu-DOTA-trastuzumab were > 98% radiochemically pure and bound HER2 with high affinity (*K*_d _= 20.4 ± 2.5 nM and 40.8 ± 3.5 nM, respectively). MDA-MB-361 and SKOV-3 tumors were most clearly imaged using ^111^In- and ^64^Cu-DOTA-trastuzumab Fab. Significantly higher tumor/blood (*T*/*B*) ratios were found for ^111^In-DOTA-trastuzumab Fab than ^111^In-DOTA-rituximab Fab for BT-20, MDA-MB-231 and MDA-MB-361 xenografts, and there was a direct association between *T*/*B *ratios and HER2 expression. In contrast, tumor uptake of ^64^Cu-DOTA-trastuzumab Fab was significantly higher than ^64^Cu-DOTA-rituximab Fab in MDA-MB-361 tumors but no direct association with HER2 expression was found. Both ^111^In- and ^64^Cu-DOTA-trastuzumab Fab imaged small (5 to 10 mm) or larger (10 to 15 mm) MDA-MB-361 tumors. Higher blood, liver, and spleen radioactivity were observed for ^64^Cu-DOTA-trastuzumab Fab than ^111^In-DOTA-trastuzumab Fab.

**Conclusions:**

We conclude that ^111^In-DOTA-trastuzumab Fab was more specific than ^64^Cu-DOTA-trastuzumab Fab for imaging HER2-positive tumors, especially those with low receptor density. This was due to higher levels of circulating radioactivity for ^64^Cu-DOTA-trastuzumab Fab which disrupted the relationship between HER2 density and *T*/*B *ratios. Use of alternative chelators that more stably bind ^64^Cu may improve the association between *T*/*B *ratios and HER2 density for ^64^Cu-labeled trastuzumab Fab.

## Background

The human epidermal growth factor receptor-2 (HER2) is overexpressed in 20% to 25% of breast cancers (BC) and is the target for treatment with trastuzumab (Herceptin), a humanized IgG_1 _monoclonal antibody (mAb) [1,2]. HER2 amplification is normally assessed *ex vivo *in a primary tumor biopsy by immunohistochemical (IHC) staining for HER2 protein or by fluoresecence *in situ *hybridization to detect increased *HER2 *gene copy number [3]. However, discordance in HER2 expression between primary and metastatic BC has been found in 20% to 30% of cases [4,5] and thus, it would be useful to have an imaging technique to assess HER2 phenotype *in situ *in BC lesions. Several investigators have shown that HER2 expression can be imaged in human BC xenografts in athymic mice by single photon emission computed tomography (SPECT) using trastuzumab or its Fab fragments labeled with ^111^In or ^99 m^Tc [6-9]. These studies have been extended to imaging HER2-positive BC in patients using ^111^In-labeled trastuzumab IgG [2,10]. More recently, positron-emission tomography (PET) using trastuzumab labeled with ^89^Zr has shown promise for imaging HER2 expression in tumor xenograft mouse models and in patients with metastatic BC [11,12]. Imaging also offers an opportunity to detect response to HER2-targeted therapies in BC [13]. We previously reported that SPECT with ^111^In-labeled pertuzumab (anti-HER2) detected early response to treatment with trastuzumab (Herceptin) in athymic mice bearing s.c. MDA-MB-361 BC xenografts [14]. Smith-Jones et al. demonstrated that PET with ^68^Ga-labeled trastuzumab F(ab')_2 _fragments identified response of HER2-positive BT-474 human BC tumors in mice to treatment with heat shock protein (Hsp90) inhibitors [15].

PET offers several potential advantages compared to SPECT for imaging tumors because it has higher intrinsic sensitivity, is more easily quantified, and in some instances offers higher spatial resolution. Despite these apparent benefits, few studies have reported a comparison of PET and SPECT for imaging HER2-positive tumors using the same agent labeled with a single photon-emitter or positron-emitter. Dijkers et al. compared ^89^Zr- and ^111^In-labeled trastuzumab in mice bearing s.c. SK-OV-3 human ovarian cancer xenografts and reported no significant differences in tumor and normal tissue uptake [12]. MicroPET with ^89^Zr-labeled trastuzumab visualized these tumors, but the corresponding microSPECT images with ^111^In-labeled trastuzumab were not presented.

In this study, we compared microSPECT/CT and microPET/CT for imaging s.c. human tumor xenografts expressing a wide range of HER2 density in athymic mice using trastuzumab Fab fragments modified with 1,4,7,10-tetraazacyclododecane N, N', N″, N'″-tetraacetic acid (DOTA) for complexing ^111^In or ^64^Cu. ^64^Cu decays with a half-life of 12.7 h by positron emission [Eβ^+ ^= 0.65 MeV (17.4%)], β^- ^emission [Eβ = 0.58 MeV (39%)] and electron capture (43.6%). ^111^In decays by electron capture with a half-life of 2.8 days emitting Auger electrons and two γ-photons [Eγ = 171 keV (90%) and 245 keV (94%)]. DOTA was selected as a chelator because both ^111^In and ^64^Cu form thermodynamically stable complexes with DOTA (K_d _= 10^24 ^and 10^23 ^M^-1^, respectively) [16,17]. ^64^Cu complexed to DOTA and linked to mAbs and peptides has been widely studied for PET imaging of tumors [15,18-23]. Fab fragments were selected for these studies because their pharmacokinetics of tumor uptake and elimination from the blood and normal tissues is compatible with the half-lives of ^64^Cu and ^111^In [24].

## Materials and methods

### Preparation of Fab fragments

Trastuzumab (Herceptin) and rituximab (anti-CD20; Rituxan) are humanized IgG_1 _mAbs and were obtained from Roche Pharmaceuticals Ltd. (Mississauga, ON, Canada). Fab fragments were prepared by digestion with immobilized papain (Pierce Chemical Co., Rockford, IL, USA) and purified as reported [7,25]. Fab purity was assessed by sodium dodecyl sulfate polyacrylamide gel electrophoresis (SDS-PAGE) on a 4% to 20% Tris HCl gradient mini-gel (BioRad, Mississauga, ON, Canada) and by size-exclusion high performance liquid chromatography (HPLC). For SDS-PAGE, Fab (10 μg) were electrophoresed under non-reducing and reducing [dithiothreitol (DTT)] conditions. The gel was stained with Coomassie R-250 brilliant blue (Bio-Rad). Size-exclusion HPLC was performed on a BioSep SEC-2000 column (Phenomenex, Torrance, CA, USA) eluted with 100 mM NaH_2_PO_4 _buffer (pH 7.0) at a flow rate of 0.8 mL/min in line with a diode array detector (PerkinElmer, Wellesley, MA, USA) monitoring at 280 nm. Fab fragments were concentrated and buffer-exchanged into 50 mM NaHCO_3 _buffer (pH 7.5) on an Amicon Ultracel 30 K device (*M_r _*cut-off = 30 kDa; Millipore Corp., Billerica, MA, USA). Trace metals were removed from all buffers using Chelex-100 cation-exchange resin (BioRad). The final Fab fragments concentration was measured spectrophotometrically [*E_280 nm _*= 1.45 (mg/mL)^-1 ^cm^-1^] [7] and was adjusted to 5 mg/mL with 50 mM NaHCO_3 _buffer, pH 7.5.

### DOTA conjugation and radiolabeling of Fab fragments

Trastuzumab or rituximab Fab fragments were modified with DOTA for complexing ^111^In or ^64^Cu by reaction of 1.5 mg of Fab in 300 μL of NaHCO_3 _buffer (pH 7.5) with a 60- or 90-fold excess, respectively, of the N-hydroxysuccinimidyl ester of 1,4,7,10-tetraazacyclododecane tetraacetic acid (NHS-DOTA; Macrocyclics, Dallas, TX, USA). The conjugation reaction was performed at 4°C for 18 h. DOTA-conjugated Fab were purified from excess DOTA by transferring to an Amicon Ultracel 30 K device, diluting to 12.0 mL with 1 M CH_3_COONH_4 _buffer, pH 6.0 and centrifuging at 4,000 × *g *for 15 min. This purification step was repeated six times. Finally, purified DOTA-Fab were recovered and the concentration determined spectrophotometrically [*E_280 nm _*= 1.45 (mg/mL)^-1 ^cm^-1^]. The final concentration was adjusted to 5 mg/mL with 1 M CH_3_COONH_4 _buffer, pH 6.0.

Radiolabeling was performed by incubating 50 μg of DOTA-Fab in 10 μL of CH_3_COONH_4 _buffer, pH 6.0 with 360 MBq of ^111^InCl_3 _(> 7 GBq/mL; MDS-Nordion, Kanata, ON, Canada) or 216 MBq of ^64^CuCl_2 _(> 4 GBq/mL; MDS-Nordion) for 3 h at 46°C. ^111^In- or ^64^Cu-labeled DOTA-Fab were purified on an Amicon Ultracel 30 K device. The final radiochemical purity was measured by instant thin layer-silica gel chromatography (ITLC-SG; Pall Life Sciences, Ann Arbor, MI, USA) developed in 100 mM sodium citrate, pH 5.0 or by size-exclusion HPLC using a flow-through radioactivity detector (FSA; PerkinElmer). The *R*_f _values for ^111^In- or ^64^Cu-DOTA-Fab on ITLC were 0.0 and those for ^111^In- or ^64^Cu-DOTA or free radionuclides were 1.0. The DOTA substitution level of the Fab fragments (chelators/molecule) was measured by labeling a 10 μL aliquot of the unpurified conjugation reaction with ^111^In, then determining the proportion of ^111^In-DOTA-Fab *vs*. free ^111^In-DOTA by ITLC-SG and multiplying this fraction by the molar ratio used in the reaction [26].

### HER2 binding affinity of ^111^In- and ^64^Cu-DOTA-trastuzumab Fab

The HER2 binding affinity of ^111^In- and ^64^Cu-DOTA-trastuzumab Fab was determined by direct (saturation) radioligand binding assays using SKBR-3 human BC cells (1.3 × 10^6 ^HER2/cell) [9]. Briefly, increasing concentrations (0 to 600 nmol/L) of ^111^In- or ^64^Cu-DOTA-trastuzumab Fab were incubated with 1 × 10^5 ^cells in 24-well plates at 4°C for 3 h. Unbound radioactivity was removed and the dishes were rinsed two times with phosphate-buffered saline. The cells were dissolved in 100 mM NaOH, recovered, and the total cell-bound radioactivity (TB) was measured in a γ-counter (PerkinElmer Wizard 3). The assay was repeated in the presence of 16 μmol/L of unlabeled trastuzumab IgG to measure non-specific binding (NSB). Specific binding (SB; nanomoles per liter) was calculated by subtracting NSB from TB and was plotted *vs*. the concentration of ^111^In- or ^64^Cu-DOTA-trastuzumab Fab (nanomoles per liter) added. The resulting curve was fitted by non-linear regression to a one-site receptor-binding model by Prism Ver. 4.0 software (GraphPad, San Diego, CA, USA). The dissociation constant (*K*_d_) and maximum number of receptors per cell (*B*_max_) were calculated and compared for ^111^In- and ^64^Cu-DOTA-trastuzumab Fab.

### Tumor and normal tissue distribution studies

The tumor and normal tissue distribution of ^111^In- or ^64^Cu-DOTA-trastuzumab Fab were determined at 24-h post-intravenous (tail vein) injection (p.i.) in athymic mice with s.c. human tumor xenografts with a wide range of HER2 density. This time point was selected due to the short physical half-life of ^64^Cu (12.7 h) and because high tumor uptake [> 5 percent injected dose per gram (% i.d./g)] and tumor/blood (*T*/*B*) ratios (> 4:1) were previously found for ^111^In-DTPA-trastuzumab Fab at 24 h p.i. [7]. Tumors were established in female athymic (CD1-nude) mice by s.c. inoculation of 1 × 10^7 ^MDA-MB-231, BT-20, or MDA-MB-361 BC cells expressing 5.4 × 10^4^, 1.6 × 10^5^, or 5.1 × 10^5 ^HER2/cell, respectively, or with SK-OV-3 ovarian cancer cells displaying 1.2 × 10^6 ^HER2/cell [27]. At 4 to 7 weeks post-inoculation, when tumors were well established (5 to 15 mm in diameter), groups of mice (*n *= 4) were injected i.v. (tail vein) with 12 MBq (10 μg) of ^111^In-DOTA-trastuzumab Fab or 18 MBq (10 μg) of ^64^Cu-DOTA-trastuzumab Fab. To determine if tumor uptake was specific, control groups of mice (*n *= 4) with MDA-MB-231, BT-20, or MDA-MB-361 xenografts were injected i.v. with 12 MBq (10 μg) of irrelevant ^111^In-DOTA-rituximab Fab or 18 MBq (10 μg) of ^64^Cu-DOTA-rituximab Fab. Mice were euthanized by cervical dislocation under general anaesthesia. Tumor and normal tissue uptake of radioactivity was measured in a γ-scintillation counter (Wizard 3, PerkinElmer, Waltham, MA) was expressed as percent injected dose per gram and as tumor/normal tissue (*T*/*NT*) ratios. The relationship between tumor/blood (*T*/*B*) ratios and HER2 density was examined. The uptake of ^111^In- or ^64^Cu-DOTA-trastuzumab Fab fragments in small (5 to 10 mm diameter) *vs*. larger (10 to 15 mm diameter) tumor xenografts was compared.

### MicroSPECT and microPET imaging

MicroSPECT was performed at 24 h p.i. of 70 MBq (10 μg) of ^111^In-DOTA-trastuzumab Fab or ^111^In-DOTA-rituximab Fab in athymic mice with s.c. HER2-positive tumor xenografts. Anaesthesia was induced and maintained by inhalation of 2% isoflurane in O_2_. MicroSPECT was performed on a NanoSPECT/CT tomograph (Bioscan, Washington, DC, USA) equipped with four NaI scintillation detectors fitted with 1.4-mm multi-pinhole collimators [full-width half-maximum (FWHM) = 1.2 mm]. A total of 24 projections were acquired in a 256 × 256 matrix with a minimum of 80,000 counts per projection. MicroSPECT image acquisition time was 85 to 120 mins. MicroSPECT images were reconstructed using an ordered-subset expectation maximization (OSEM) algorithm (nine iterations). Prior to microSPECT imaging, cone-beam CT images were acquired (180 projections, 1 s/projection, 45 kVp) on the NanoSPECT/CT system. Co-registration of microSPECT and CT images was performed using InvivoScope software (Bioscan).

MicroPET was performed at 24 h p.i. of 22 MBq (10 μg) of ^64^Cu-DOTA-trastuzumab Fab or ^64^Cu-DOTA-rituximab Fab on a Focus 220 microPET tomograph (Siemens Preclinical Solutions, Knoxville, TN, USA). Images were acquired for 20 mins and reconstructed using OSEM, followed by a maximum *a posteriori *probability reconstruction algorithm with no correction for attenuation or partial-volume effects. The FWHM resolution of the microPET tomograph was 1.6 mm. Immediately after imaging, CT was performed on an eXplore Locus Ultra Preclinical CT scanner (GE Healthcare, Mississauga, ON, Canada) with routine acquisition parameters (80 kVp, 70 mA, and voxel size of 150 × 150 × 150 mm). MicroPET and CT images were coregistered using Inveon Research Workplace software (Siemens). All animal studies were conducted under a protocol (no. 989.9) approved by the Animal Use Committee at the University Health Network following Canadian Council on Animal Care guidelines.

### Statistical analyses

Statistical significance of comparisons were assessed by Student's *t *test (*P *< 0.05).

## Results

### Preparation of ^111^In and ^64^Cu-labeled DOTA-Fab fragments

**Figure 1:**
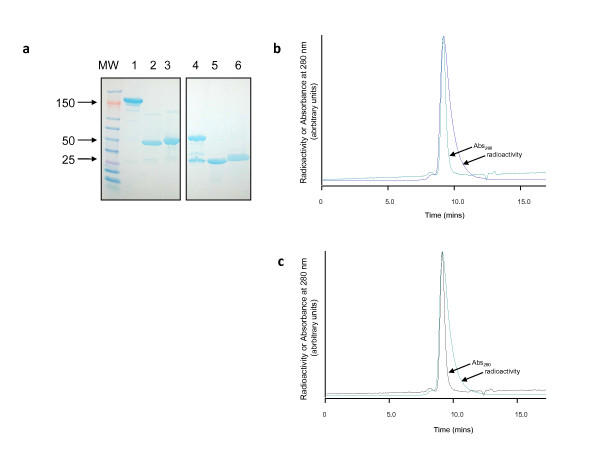
**SDS-PAGE and size-exclusion HPLC**. **(a) **SDS-PAGE analysis on a 4% to 20% Tris HCl gradient mini-gel stained with Coomassie Brilliant Blue of trastuzumab (lane 1), trastuzumab Fab (lane 2), and DOTA-trastuzumab Fab (lane 3) under non-reducing conditions or these same proteins under reducing conditions (lanes 4 to 6, respectively). Molecular weight markers are shown (lane MW). The positions of 150, 50, and 25 kDa markers are indicated. Size-exclusion HPLC analyses of (**b**) ^111^In-DOTA-trastuzumab Fab (top panel) and (**c**) ^64^Cu-DOTA-trastuzumab Fab (bottom panel) with detection of absorbance at 280 nm or radioactivity.

SDS-PAGE (Figure 1a) and size-exclusion HPLC (Figure 1b,c) demonstrated that pure (> 98%) Fab fragments of trastuzumab and rituximab were obtained by digestion of intact IgG_1 _with immobilized papain using a previously reported method [7,25]. Reaction of trastuzumab and rituximab Fab with a 60- or 90-fold excess of NHS-DOTA for 18 h at 4°C resulted in substitution of 3.7 ± 0.2 and 2.5 ± 0.3 DOTA chelators per molecule, respectively. The pre-purification labeling efficiencies for ^111^In-DOTA-trastuzumab Fab, ^111^In-DOTA-rituximab Fab, ^64^Cu-DOTA-trastuzumab Fab, and ^64^Cu-DOTA-rituximab Fab were 75.5 ± 5.4%, 76.8 ± 1.5%, 65.9 ± 4.9%, and 67.9 ± 5.7%, respectively. Following purification, the radiochemical purity was > 98% for all radioimmunoconjugates by ITLC (not shown) and size-exclusion HPLC (Figure 1b,c). The specific activities of ^111^In- and ^64^Cu-DOTA-trastuzumab Fab fragments used in microSPECT and microPET and biodistribution studies were 3.6 to 4.9 MBq/μg and 1.3 to 4.7 MBq/μg, respectively. The specific activities of ^111^In- and ^64^Cu-DOTA-rituximab Fab were 1.3 to 5.8 and 1.9 to 2.8 MBq/μg.

### HER2 binding affinity of ^111^In- and ^64^Cu-DOTA-trastuzumab Fab

**Figure 2:**
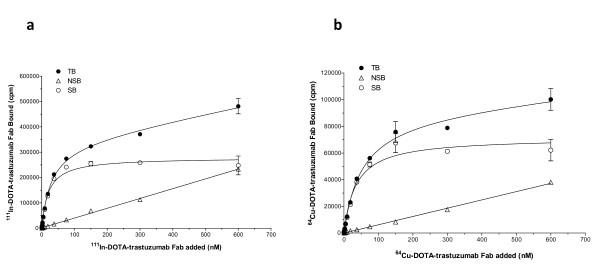
**Direct (saturation) radioligand binding assays**. Direct (saturation) radioligand binding to SKBR-3 human breast cancer cells of **(a) **^111^In-DOTA-trastuzumab Fab or **(b) **^64^Cu-DOTA-trastuzumab Fab, in the absence (total binding; TB) or presence (non-specific binding; NSB) of excess (16 μM) unlabeled trastuzumab IgG. Specific binding (SB) was calculated by subtraction of NSB from TB. Curves were fitted to a 1-site receptor-binding model using Prism Ver. 4.0 software (GraphPad).

Direct (saturation) radioligand binding assays showed that ^111^In- and ^64^Cu-DOTA-trastuzumab Fab bound specifically to HER2 on SKBR-3 cells (Figure 2a,b). The *K*_d _values for ^111^In- and ^64^Cu-DOTA-trastuzumab Fab were 20.4 ± 2.5 nM and 40.8 ± 3.5 nM (*P *< 0.01), respectively. These values were similar to the *K*_d _for ^111^In-DTPA-trastuzumab Fab binding to SKBR-3 cells previously reported by our group (*K*_d _= 48 nM) [25]. There was no specific binding of ^111^In-DOTA-rituximab to SKBR-3 cells (not shown). The *B*_max _values for ^111^In- and ^64^Cu-DOTA-trastuzumab Fab on SKBR-3 cells were 1.4 ± 0.1 × 10^6 ^receptors/cell and 2.3 ± 0.1 × 10^6 ^receptors/cell, respectively (*P *< 0.001).

### Tumor and normal tissue distribution studies

**Figure 3:**
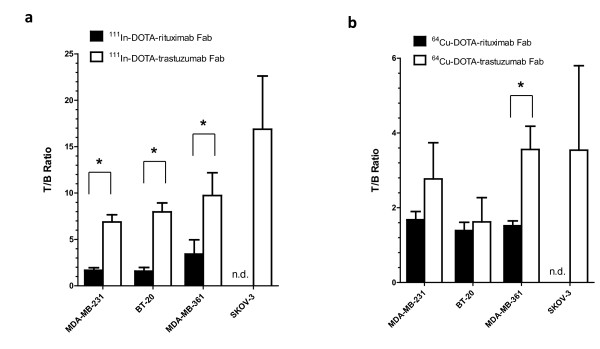
**Tumor/blood (*T*/*B*) ratios**. *T/B *ratios at 24 h p.i. of **(a) **^111^In-DOTA-trastuzumab Fab and ^111^In-DOTA-rituximab Fab or **(b) **^64^Cu-DOTA-trastuzumab Fab and ^64^Cu-DOTA-rituximab Fab, in s.c. human tumor xenografts in athymic mice with increasing HER2 expression. Values shown are mean ± SD (*n *= 4). The HER2 density (receptors/cell) was 5.4 × 10^4 ^(MDA-MB-231), 1.6 × 10^5 ^(BT-20), 5.1 × 10^5 ^(MDA-MB-361) and 1.2 × 10^6 ^(SK-OV-3). Significant differences (*P *< 0.05) between ^111^In- or ^64^Cu-labeled trastuzumab Fab and rituximab Fab are indicated by asterisks. n.d., not determined.

The tumor and normal tissue uptake of ^111^In- and ^64^Cu-DOTA-trastuzumab Fab at 24 h p.i. in athymic mice bearing s.c. MDA-MB-361 human BC xenografts (5.1 × 10^5 ^HER2/cell) are shown in Table 1. Blood levels were threefold significantly higher for ^64^Cu- than ^111^In-DOTA-trastuzumab Fab (1.40 ± 0.16% *vs*. 0.42 ± 0.08% i.d./g; *P *< 0.0001). Similarly, liver uptake was threefold significantly greater for ^64^Cu- than ^111^In-DOTA-trastuzumab Fab (8.52 ± 0.81% *vs*. 3.13 ± 0.15% i.d./g; *P *< 0.0001). Radioactivity concentrations were higher in heart, lungs, stomach, intestines, and spleen for ^64^Cu- than ^111^In-DOTA-trastuzumab Fab (Table 1). However, kidney uptake was not significantly different between ^64^Cu- and ^111^In-DOTA-trastuzumab Fab (57.00 ± 7.09% *vs*. 62.85 ± 6.45% i.d./g; *P *= 0.268). There was no significant difference in tumor accumulation for ^111^In- and ^64^Cu-DOTA-trastuzumab Fab (4.00 ± 0.90% *vs*. 5.00 ± 1.2% i.d./g; *P *= 0.228). Due to the higher blood and liver radioactivity, the *T*/*B *and tumor/liver (*T*/*L*) ratios were three- and twofold significantly lower, respectively for ^64^Cu- than ^111^In-DOTA-trastuzumab Fab (3.56 ± 0.62 *vs*. 9.73 ± 2.46; *P *= 0.003 and 0.59 ± 0.16 *vs*. 1.27 ± 0.26 *vs*.; *P *= 0.004, respectively; Table 2). *T*/*NT *ratios for ^64^Cu-DOTA-trastuzumab Fab were significantly lower than ^111^In-DOTA-trastuzumab Fab for all tissues except kidneys and muscle (Table 2). There was no significant difference in the uptake of ^111^In- or ^64^Cu-DOTA-trastuzumab Fab in small (5 to 10 mm diameter) *vs*. larger (10 to 15 mm) MDA-MB-361 tumor xenografts (4.00 ± 0.91% *vs*. 6.12 ± 0.84% i.d./g; *P *= 0.138 and 5.01 ± 1.20% *vs*. 7.12 ± 1.67% i.d./g, *P *= 0.342, respectively). Absolute tumor uptake was not informative on the relationship between tumor localization of the radioimmunoconjugates and HER2 expression. Tumor uptake of ^111^In-DOTA-trastuzumab in MDA-MB-231, BT-20, MDA-MB-361, or SKOV-3 xenografts with increasing HER2 density was 4.7 ± 0.6%, 5.5 ± 0.7%, 4.0 ± 0.9%, and 5.4 ± 0.4% i.d./g. Tumor uptake of ^64^Cu-DOTA-trastuzumab in MDA-MB-231, BT-20, MDA-MB-361, or SKOV-3 xenografts was 4.4 ± 1.6%, 2.6 ± 1.8%, 5.0 ± 1.2%, and 5.0 ± 3.0% i.d./g. However, there was a strong direct relationship between *T*/*B *ratios for ^111^In-DOTA-trastuzumab Fab and tumor HER2 density (Figure 3a). Moreover, the *T*/*B *ratios for ^111^In-DOTA-trastuzumab Fab were significantly greater than irrelevant ^111^In-DOTA-rituximab Fab for MDA-MB-231, BT-20, and MDA-MB-361 xenografts, demonstrating specific localization. The *T*/*B *ratios for ^64^Cu-DOTA-trastuzumab Fab were significantly greater than ^64^Cu-DOTA-rituximab Fab for MDA-MB-361 tumors with high HER2 density (*P *< 0.001), but not for MDA-MB-231 or BT-20 xenografts with low HER2 expression (*P *= 0.0709 and 0.528, respectively; Figure 3b). The localization of ^111^In- or ^64^Cu-DOTA-rituximab Fab in SK-OV-3 tumors was not determined. No relationship between the *T*/*B *ratios for ^64^Cu-DOTA-trastuzumab Fab and HER2 density was established (Figure 3b).

**Table 1:** Tumor and normal tissue distribution at 24 h post-injection of ^111^In- or ^64^Cu-DOTA-trastuzumab Fab

	Percent injected dose/g ^a, b, c^	
Tissue	^111^In-DOTA-trastuzumab Fab	^64^Cu-DOTA-trastuzumab Fab
Blood	0.42 ± 0.08	1.40 ± 0.16
Heart	0.92 ± 0.06	2.42 ± 0.39
Lungs	0.80 ± 0.14	4.86 ± 0.57
Liver	3.13 ± 0.16	8.52 ± 0.81
Kidneys	62.85 ± 6.45	57.00 ± 7.09
Stomach	0.58 ± 0.05	3.41 ± 0.22
Intestines	0.61 ± 0.06	4.76 ± 0.41
Spleen	2.25 ± 0.13	5.00 ± 0.27
Muscle	0.78 ± 0.31	0.75 ± 0.06
Tumor	4.00 ± 0.91	5.01 ± 1.20

^111^In- or ^64^Cu-DOTA-trastuzumab Fab in athymic mice bearing subcutaneous MDA-MB-361 human breast cancer xenografts. ^a^Values shown are mean ± SD (*n *= 4). ^b^Significantly different for ^111^In- and ^64^Cu-DOTA-trastuzumab Fab for blood, heart, lungs, liver, stomach, intestines, and spleen (all *P *< 0.001). ^c^Not significantly different for ^111^In- and ^64^Cu-DOTA-trastuzumab Fab for kidneys, muscle, or tumor (*P *> 0.05).

**Table 2:** Tumor/normal tissue (T/NT) ratios at 24 h post-injection of ^111^In- or ^64^Cu-DOTA-trastuzumab Fab

	T/NT Ratio ^a, b, c^	
Tissue	^111^In-DOTA-trastuzumab Fab	^64^Cu-DOTA-trastuzumab Fab
Blood	9.73 ± 2.46	3.56 ± 0.62
Heart	4.31 ± 0.83	2.09 ± 0.56
Lungs	5.06 ± 1.12	1.03 ± 0.19
Liver	1.27 ± 0.26	0.59 ± 0.16
Kidneys	0.06 ± 0.02	0.09 ± 0.02
Stomach	6.99 ± 2.05	1.48 ± 0.37
Intestines	6.61 ± 1.78	1.05 ± 0.22
Spleen	1.79 ± 0.48	1.00 ± 0.20
Muscle	6.12 ± 3.33	6.78 ± 1.90

^111^In- or ^64^Cu-DOTA-trastuzumab Fab in athymic mice bearing subcutaneous MDA-MB-361 human breast cancer xenografts. ^a^Values shown are mean ± SD (*n *= 4). ^b^Significantly different for ^111^In- and ^64^Cu-DOTA-trastuzumab Fab for lungs and intestines (both *P *< 0.001), blood, heart, liver, stomach (all *P *< 0.01) and spleen (*P *< 0.05). ^c^Not significantly different for ^111^In- and ^64^Cu-DOTA-trastuzumab Fab for kidneys and muscle (*P *> 0.05).

### MicroSPECT/CT and microPET/CT imaging

**Figure 4:**
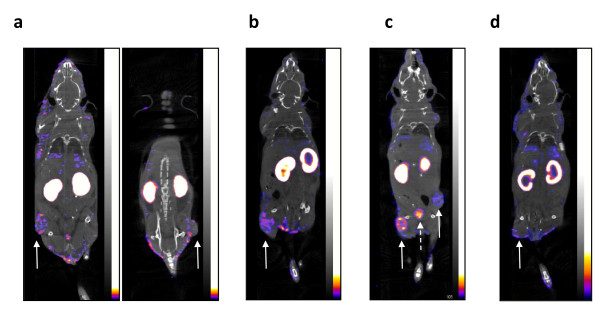
**Coronal slice microSPECT/CT images**. Images of athymic mice with s.c. human tumor xenografts (solid arrows) with increasing HER2 expression at 24 h p.i. of 70 MBq (10 μg) of ^111^In-DOTA-trastuzumab [panels (**a**), (**b**), (**c**)] or ^111^In-DOTA-rituximab Fab [panel (**d**)]. **(a) **Mouse with MDA-MB-231 (left panel) and BT-20 (right panel) xenografts with low HER2 density (5.4 × 10^4 ^and 1.6 × 10^5 ^receptors/cell, respectively). **(b) **Mouse with MDA-MB-361 xenograft with intermediate HER2 density (5.1 × 10^5 ^receptors/cell). **(c) **Mouse with 15 to 18 mm diameter (left flank) and 5 to 10 mm diameter (right flank) SK-OV-3 xenografts with high HER2 density (1.2 × 10^6 ^receptors/cell). **(d) **Mouse with MDA-MB-361 xenograft with intermediate HER2 density (5.1 × 10^5 ^receptors/cell). Bladder radioactivity in panel (c) is indicated by broken arrow. Image acquisition time was 85 to 120 min and anaesthesia was induced and maintained by inhalation of 2% isoflurane in oxygen. Images were adjusted to approximately equal intensity.

**Figure 5:**
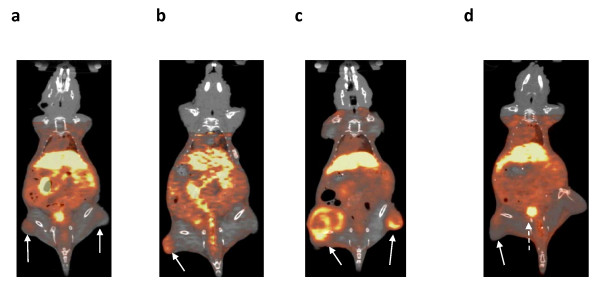
**Coronal slice microPET/CT images**. Images of athymic mice with s.c. human tumor xenografts (arrows) with increasing HER2 expression at 24 h p.i. of 22 MBq (10 μg) ^64^Cu-DOTA-trastuzumab [panels (**a**), (**b**), (**c**)] or ^64^Cu-DOTA-rituximab Fab [panel (**d**)]. **(a) **Mouse with MDA-MB-231 and BT-20 xenografts implanted on the left and right flanks, respectively with low HER2 density (5.4 × 10^4 ^and 1.6 × 10^5 ^receptors/cell, respectively). **(b) **Mouse with MDA-MB-361 xenograft with intermediate HER2 density (5.1 × 10^5 ^receptors/cell). **(c) **Mouse with 15 to 18 mm diameter (left) and 5 to 10 mm diameter (right) SK-OV-3 xenografts with high HER2 density (1.2 × 10^6 ^receptors/cell). **(d) **Mouse with MDA-MB-361 xenograft with intermediate HER2 density (5.1 × 10^5 ^receptors/cell). Bladder radioactivity seen in one mouse is indicated by a broken arrow. Image acquisition time was 20 min and anaesthesia was induced and maintained by inhalation of 2% isoflurane in oxygen. Images were adjusted to approximately equal intensity.

**Figure 6:**
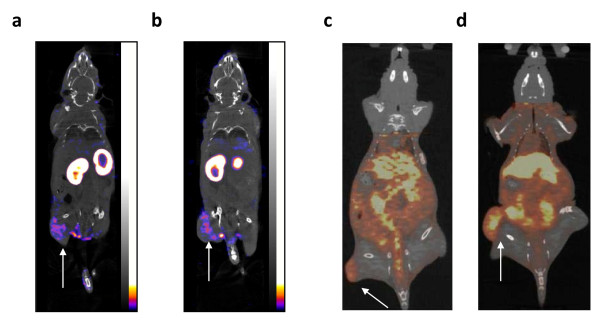
**Coronal slice microSPECT/CT [(a) and (b)] or microPET/CT images [(c) and (d)]**. Athymic mice with s.c. MDA-MB-361 tumor xenografts (solid arrows) with intermediate HER2 density (5.1 × 10^5 ^receptors/cell) at 24 h p.i. of 70 MBq (10 μg) of ^111^In-DOTA-trastuzumab Fab or 22 MBq (10 μg) of ^64^Cu-DOTA-trastuzumab Fab, respectively. **(a) **and **(c) **Mouse with 5 to 10 mm diameter tumor. **(b) **and **(d) **Mouse with 10 to 15 mm diameter tumor. Image acquisition time was 85 to 120 min for microSPECT and 20 min for microPET. Anaesthesia was induced and maintained by inhalation of 2% isoflurane in oxygen. Images were adjusted to approximately equal intensity within each modality.

Representative microSPECT and microPET images of athymic mice with s.c. tumor xenografts with increasing HER2 density at 24 h p.i. of ^111^In- or ^64^Cu-DOTA-trastuzumab Fab, respectively are shown in Figures 4 and 5. MicroSPECT/CT and microPET/CT images were displayed as coronal slices with the plane selected to optimally display the tumor uptake of ^111^In-DOTA-trastuzumab Fab or ^64^Cu-DOTA-trastuzumab Fab. MDA-MB-231 tumors with low HER2 expression (5.4 × 10^4 ^receptors/cell; Figures 4a and 5a) were least intensely imaged while SK-OV-3 tumors with high HER2 density (1.2 × 10^6 ^receptors/cell) were most clearly seen (Figures 4c and 5c) with ^111^In- or ^64^Cu-DOTA-trastuzumab. An intermediate tumor signal was found for MDA-MB-361 xenografts with 5.1 × 10^5 ^HER2/cell (Figures 4b and 5b). The specificity of tumor localization of ^111^In- or ^64^Cu-DOTA-trastuzumab Fab was shown by the lower accumulation of ^111^In- or ^64^Cu-DOTA-rituximab Fab on images of mice bearing MDA-MB-361 xenografts (Figures 4d and 5d). There was no difference in the ability of ^111^In- or ^64^Cu-DOTA-trastuzumab Fab to image small (5 to 10 mm; Figure 6a,c) or larger (10 to 15 mm; Figure 6b, d) MDA-MB-361 tumors. The kidneys were most prominent on microSPECT images of ^111^In-DOTA-trastuzumab Fab, while microPET with ^64^Cu-DOTA-trastuzumab Fab showed high liver and kidney uptake.

## Discussion

Our results revealed that both microSPECT/CT and microPET/CT with ^111^In- or ^64^Cu-DOTA-trastuzumab Fab fragments were able to image s.c. human tumor xenografts in mice with low, intermediate, or high HER2 expression. The range of HER2 expression examined (5.4 × 10^4 ^to 1.2 × 10^6 ^receptors/cell) corresponded to HER2 scores of 0 to 3+ assessed clinically in BC specimens by IHC staining [9]. There was no apparent increased ability of microPET/CT with ^64^Cu-DOTA-trastuzumab Fab compared to microSPECT/CT with ^111^In-DOTA-trastuzumab Fab to visualize MDA-MB-231 tumors with low HER2 density (1.6 × 10^5 ^receptors/cell; Figures 4a and 5a). In addition, there was no increased ability of microPET/CT using ^64^Cu-DOTA-trastuzumab Fab to image small (5 to 10 mm diameter) or larger (10 to 15 mm diameter) MDA-MB-361 tumors with intermediate HER2 expression (5.1 × 10^5 ^HER2/cell; Figure 6). The intensity of the tumor signal was dependent on HER2 expression with tumors with intermediate (MDA-MB-361) or high (SK-OV-3) HER2 density most readily imaged by microSPECT/CT (Figure 4) or microPET/CT (Figure 5). However, a threefold higher dose of radioactivity was administered for microSPECT/CT than microPET/CT (70 *vs*. 22 MBq) and image acquisition times were up to sixfold longer for microSPECT/CT (85 to 20 *vs*. 20 min, respectively). Thus, the photon detection efficiency (i.e., intrinsic sensitivity) was much higher for microPET/CT than microSPECT/CT. Nonetheless, our results revealed that provided that the administered dose of radioactivity was sufficient and image acquisition times were long enough to yield good counting statistics, microSPECT/CT with ^111^In-DOTA-trastuzumab Fab was able to image tumors with the similar HER2 density and size as microPET/CT with ^64^Cu-DOTA-trastuzumab. These results agree with those reported by Cheng et al. who noted that s.c. HER2-positive SUM190 tumor xenografts were imaged by either microSPECT or microPET using trastuzumab conjugated to biotinylated ^99 m^Tc- or ^18^F-labeled phosphodiamidate morpholinos (MORFs) through a streptavidin linker [28]. The doses of ^99 m^Tc or ^18^F used in their study were 13 and 0.22 MBq, respectively. Phantom studies revealed that microPET was 15-fold more sensitive in terms of photon detection, but the spatial resolution of microSPECT was superior to that of microPET (1.2 *vs*. 2.4 mm). The results are also in concordance with those reported by Wong et al. [29], who showed that s.c. epidermal growth factor receptor-positive LS174T human colon cancer xenografts could be imaged using panitumomab F(ab')_2 _fragments labeled with ^111^In or ^86^Y. However, they compared low resolution planar γ-camera imaging with microPET. In our study, we used similar quality high resolution and high sensitivity small animal imaging technologies, namely microSPECT/CT (NanoSPECT; Bioscan) and microPET (Siemens Focus 220) systems for these comparisons.

*T*/*B *ratios were used to compare the tumor localization of ^111^In- and ^64^Cu-DOTA-trastuzumab Fab *vs*. HER2 density because we previously found that there are differences in perfusion between different tumor xenografts which can affect the uptake of radioimmunoconjugates [9]. Use of *T*/*B *ratios minimizes these effects by normalizing for blood concentrations which then reveals the relationships between HER2 density and tumor accumulation. Moreover, the *T*/*B *ratios are important for discriminating tumors that have different HER2 expression on the images. There was a strong and direct association between the *T*/*B *ratios for ^111^In-DOTA-trastuzumab and tumor HER2 density (Figure 3a). In addition, the *T*/*B *ratios for ^111^In-DOTA-trastuzumab Fab were significantly greater than those of irrelevent control ^111^In-DOTA-rituximab Fab for MDA-MB-231, BT-20, and MDA-MB-361 xenografts, demonstrating specific localization in tumors with low or intermediate HER2 expression. In contrast, specific uptake of ^64^Cu-DOTA-trastuzumab Fab was shown in MDA-MB-361 tumors with intermediate HER2 density but not for tumors with lower HER2 expression (Figure 3b). Tumor uptake was not significantly different for ^64^Cu- and ^111^In-DOTA-trastuzumab Fab, but blood radioactivity was threefold lower for ^111^In-DOTA-trastuzumab Fab (Table 1). Thus, *T*/*B *ratios were threefold lower for ^64^Cu- than ^111^In-DOTA-trastuzumab (3.6:1 *vs*. 10:1; Table 2) in mice with MDA-MB-361 tumors. The increased circulating radioactivity for ^64^Cu-DOTA-trastuzumab Fab may be due to kinetic instability of the ^64^Cu-DOTA complex with transchelation of released ^64^Cu to copper binding proteins (e.g., albumin, ceruloplasmin, or superoxide dismutase) [26]. These ^64^Cu-labeled proteins may non-specifically localize in tumors, disrupting the association between *T*/*B *ratios and HER2 density, especially for tumors with low HER2 expression (i.e., MDA-MB-231 and BT-20).

DOTA forms thermodynamically stable complexes with copper (*K*_d _= 10^23 ^M^-1^) but kinetic instability of ^64^Cu-DOTA complexes *in vivo *can lead to loss of radiometal resulting in high blood radioactivity and liver and spleen uptake [17]. In addition to the higher levels of blood radioactivity, we found that the liver and spleen uptake for ^64^Cu-DOTA-trastuzumab Fab were three- and twofold greater, respectively, than ^111^In-DOTA-trastuzumab Fab (Table 1). In order to improve the kinetic stability of ^64^Cu complexes, more thermodynamically stable cross-bridged (CB-DO2A) or sarcophagine (SarAr) chelators have been synthesized [30,31]. A comparison of ^64^Cu complexed to DOTA or CB-DO2A (but not conjugated to mAbs) showed fourfold lower radioactivity in the blood and twofold lower liver accumulation at 24 h p.i. in rats [30]. Voss et al. noted that ch14.18 mAbs labeled with ^64^Cu through the extremely stable SarAr chelator for PET imaging of neuroblastoma or melanoma xenografts in mice exhibited low liver uptake (5% to 10% i.d./g) but no comparison with other chelators was provided [31]. Dearling et al. recently compared the tumor and normal tissue distribution of these same ^64^Cu-labeled ch14.18 mAbs using a variety of chelators including DOTA and SarAr in mice bearing M21 melanoma xenografts [17]. Unexpectedly, no significant differences in tumor or liver uptake were found for ch14.18 labeled with ^64^Cu using DOTA or the much more stable SarAr chelator. They suggested that in addition to ^64^Cu-chelator stability, factors such as the net charge on the chelators may play an important role in sequestration of radioactivity by tissues. In our study, tumor uptake was not significantly different between ^111^In- and ^64^Cu-DOTA-trastuzumab Fab in mice with MDA-MB-361 tumors, despite the apparent instability of ^64^Cu-DOTA-trastuzumab Fab as evidenced by higher levels of radioactivity in the blood, liver, and spleen (Table 1). The use of more stable chelators such as CB-DO2A or SarAr may diminish blood radioactivity and improve the association between tumor HER2 density and *T*/*B *ratios for ^64^Cu-labeled trastuzumab Fab. The CB-DO2A and SarAr chelators are unfortunately not yet commercially available in a chemically reactive form for conjugation to mAbs for ^64^Cu labeling.

## Conclusion

Provided that administered doses of radioactivity and acquisition times were sufficient to yield good counting statistics, we conclude that either microSPECT/CT with ^111^In-DOTA-trastuzumab Fab or microPET/CT with ^64^Cu-DOTA-trastuzumab Fab visualized small (5 to 10 mm diameter) or larger (10 to 15 mm diameter) s.c. tumor xenografts with low, intermediate, or high HER2 expression in athymic mice. However, due to the higher levels of circulating radioactivity for ^64^Cu-DOTA-trastuzumab Fab, no association between HER2 density and *T*/*B *ratios was established. In contrast, there was a strong direct association between *T*/*B *ratios and HER2 density of these tumors for ^111^In-DOTA-trastuzumab Fab. Thus, ^111^In-DOTA-trastuzumab Fab was more specific than ^64^Cu-DOTA-trastuzumab Fab for imaging HER2-positive tumors with low HER2 density. The use of more stable CB-DO2A or SarAr chelators for ^64^Cu may potentially diminish blood radioactivity, provide a stronger association between *T*/*B *ratios and tumor HER2 density, and improve the specificity of imaging with ^64^Cu-labeled trastuzumab Fab.

## Competing interests

The authors declare that they have no competing interests.

## Authors' contributions

CC and SS synthesized the ^111^In- and ^64^Cu-DOTA-trastuzumab Fab fragments and performed characterization studies. KM and DAS performed microSPECT and microPET imaging studies. RMR wrote the manuscript with the assistance of all authors.
